# Drug-Drug Interaction Knowledge, Practices, and Barriers in Community Pharmacies: A Cross-Sectional Study from Jazan Region, Saudi Arabia

**DOI:** 10.3390/pharmacy14010012

**Published:** 2026-01-23

**Authors:** Moaddey Alfarhan, Muath F. Haqwi, Abdulrahman H. Musayyikh, Jala Ashqar, Lama Y. Suwidi, Amal H. Fageh, Enas A. Alajam, Hadi Almansour, Thamir M. Alshammari, Saeed Al-Qahtani

**Affiliations:** 1 Department of Clinical Practice, College of Pharmacy, Jazan University, Jazan 82912, Saudi Arabia; halmansour@jazanu.edu.sa (H.A.); talshammari@jazanu.edu.sa (T.M.A.); saqahtani@jazanu.edu.sa (S.A.-Q.); 2 Pharmacy Practice Research Unit, College of Pharmacy, Jazan University, Jazan 82912, Saudi Arabia; 3 College of Pharmacy, Jazan University, Jazan 82912, Saudi Arabia

**Keywords:** drug–drug interactions, community pharmacists, community pharmacies, medication safety, Saudi Arabia

## Abstract

(1) Background: Drug–drug interactions (DDIs) are a frequent cause of medication-related harm, particularly in ambulatory care. Community pharmacists are uniquely positioned to identify and manage these risks. This study assessed DDI knowledge, practices, and barriers among community pharmacists in the Jazan Region, Saudi Arabia. (2) Methods: A structured, self-administered questionnaire was distributed to community pharmacists. The survey assessed DDI knowledge using 26 clinically relevant drug pairings and included questions on professional behavior, training exposure, software use, and educational needs. Descriptive and inferential statistics were applied to identify associations between knowledge scores and demographic or practice-related variables. (3) Results: A total of 219 pharmacists participated in the study. The mean knowledge score was (9.63 ± 4.58) out of 26, reflecting suboptimal to moderate awareness. Female pharmacists demonstrated significantly higher DDI knowledge scores than males (10.74 ± 5.4 vs. 9.08 ± 4.2; *p* = 0.016). Knowledge scores also differed significantly by academic qualification (*p* < 0.001), with PharmD holders scoring higher than B. Pharm and postgraduate degree holders. Pharmacists with less than 10 years of experience had significantly higher scores compared with those with longer practice duration (*p* = 0.002). Additionally, pharmacists who graduated from Saudi institutions scored higher than those trained outside Saudi Arabia (10.22 ± 4.7 vs. 8.44 ± 4.2; *p* = 0.005). Pharmacists who had received professional development training and those who attended workshops regularly also scored significantly higher. Familiarity with guidelines showed a positive trend. Reported barriers to effective DDI counseling included time constraints, limited patient understanding, and poor collaboration with prescribers. Self-rated awareness of DDIs was positively associated with actual knowledge scores. Pharmacists expressed strong preferences for workshops, online courses, and webinars as future training formats. (4) Conclusions: Pharmacists in the Jazan Region demonstrate moderate awareness of DDIs, with variation influenced by training, experience, and qualifications. Enhancing access to structured professional development and integrating clinical decision support tools could strengthen pharmacists’ role in preventing DDIs in community practice.

## 1. Introduction

Drug–drug interactions (DDIs) are a significant contributor to medication-related harm, and a substantial proportion are preventable; DDIs account for approximately 26% of all adverse drug events (ADEs) [1]. DDIs are a significant cause of adverse drug reactions (ADRs), which can vary from mild side effects to life-threatening conditions [2]. They increase the risk of emergency visits and hospital admissions, prolong hospital stays, and raise costs [2,3,4]. The World Health Organization estimates that the annual cost of medication errors reaches $42 billion USD [5]. They are a key focus of the Global Patient Safety Challenge, Medication Without Harm, which aims to reduce severe, avoidable medication-related harm by 50% globally [5]. Potential DDIs are common in ambulatory care and community pharmacy, particularly in the context of multimorbidity and polypharmacy. Recent outpatient and community-based studies suggest that potential DDI prevalence estimates range from about one-third to over half of prescriptions in certain high-risk groups. However, these estimates differ based on patient populations, drug classes, and the sources referenced for DDIs [2,6]. Polypharmacy refers to a patient taking multiple medications (often five or more) at the same time. This practice in community pharmacy increases the risk of drug interactions and results in adverse health outcomes [7,8]. DDIs carry a considerable economic burden, reinforcing the need for robust prevention strategies across care settings.

Community pharmacists are strategically positioned to intercept DDIs through medication history taking, prospective review, clinical decision support, medication reconciliation, and patient counselling. Evidence syntheses indicate that interventions delivered by pharmacists can lower medication errors and unplanned healthcare visits. However, the extent of these effects varies based on local workflows, access to reliable drug interaction (DDI) references, and the strength of interprofessional communication channels [9,10]. Simultaneously, community pharmacy practice encounters several challenges, including limited patient-specific data, a high workload and competing priorities, poorly tailored computer alerts, and underreporting of OTC medications and supplements. These factors prevent pharmacists from effectively assessing potential interactions, which can lead to gaps in patient care and medication safety [11].

In Saudi Arabia, the health system transformation under Vision 2030 aims to strengthen patient-centered, safety-focused care and expand pharmacists’ clinical roles across various settings, including community pharmacies [12,13]. Despite this policy momentum, the literature reveals uneven knowledge of DDIs among Saudi community pharmacists. Studies from different areas in Saudi Arabia reported below-optimal accuracy across multiple interaction pairs, with only a minority correctly identified by most of the respondents [14,15]. Additional research on pharmacists’ knowledge, attitudes, and practices regarding DDIs in Saudi Arabia shows the importance of understanding how skills, tools, and system factors influence DDI management [16]. Moreover, few investigations integrate objective knowledge assessment with practice behaviors, perceived barriers, and guideline familiarity in the same cohort, limiting actionable insight for targeted quality improvement.

Jazan is a rapidly growing region with expanding community pharmacy services, but there is limited published data on DDI decision-making among community pharmacists. To address this gap, we conducted a cross-sectional study to (i) evaluate community pharmacists’ knowledge of clinically important DDIs in the Jazan Region; (ii) explore perceptions, behaviors, and system-level barriers related to DDI management; (iii) analyze demographic and training factors associated with knowledge. By combining item performance with workflow and systems data, our aim is to enhance and refine reference tools and clinical decision support, ultimately reducing preventable medication harm from DDIs in community settings.

## 2. Materials and Methods

### 2.1. Study Design and Setting

A cross-sectional study was conducted from March to June 2025 in the Jazan region of Saudi Arabia. Our study looked at pharmacists working in community pharmacies. We collected data through online questionnaires (File S1) and conducted semi-structured interviews with some participants to gather more detailed insights. The manuscript follows the STROBE reporting guidelines, and the completed checklist is available in the Supplementary Materials (Table S1).

### 2.2. Study Population

The study population consisted of licensed pharmacists employed in community pharmacies throughout the Jazan region. Eligible participants were those actively practicing in community pharmacies within the region and who provided informed consent to participate. Pharmacists practicing outside of Jazan, as well as individuals who declined or withdrew from the study, were excluded.

### 2.3. Sample Size and Sampling Procedure

According to the latest Ministry of Health statistics (2023), 999 pharmacists were working in the private sector in the Jazan region [17]. This figure was used as the target population, as official data provide pharmacist-level counts for the private sector, which includes community pharmacy practice. The required sample size was calculated using the Raosoft online sample size calculator, assuming a 90% confidence interval, a 5% margin of error, and an assumed response distribution of *p* = 0.5 to account for maximum variability, given the absence of prior local studies. Based on these assumptions, the recommended minimum sample size was 214 participants. A convenience sampling method was employed, and data collection continued until the minimum required sample size was achieved. A total of 219 community pharmacists participated in the study and were included in the final analysis.

### 2.4. Data Collection Tool and Procedure

The data collection tool was a structured questionnaire developed following a comprehensive review of the literature on pharmacists’ knowledge, attitudes, and practices related to DDIs [14,15,18,19,20,21,22,23]. The questionnaire was reviewed by academic pharmacy practice experts to guarantee its content validity. A pilot study involving 15 community pharmacists was conducted to assess clarity, relevance, and completion time, thereby ensuring face validity. Necessary modifications were incorporated before final distribution.

The 26 drug–drug interaction pairs included in the knowledge were selected based on international and regional literature assessing clinically significant drug–drug interactions (DDIs). The list of drug pairs was informed by high-priority DDI frameworks proposed by Malone et al. (2004) and Phansalkar et al. (2012) [18,19], alongside relevant studies conducted in Saudi Arabia, resulting in the selection of 26 clinically relevant interactions [14,15]. These drug pairs represent clinically significant and commonly encountered interactions, encompassing contraindicated combinations, interactions requiring close monitoring, and combinations with minimal or no interaction, allowing comparability with prior research and supporting the validity of the instrument.

Data collection was conducted on-site in community pharmacies using one of two standardized approaches, depending on the participant’s preference. Pharmacists who agreed to participate verbally completed the questionnaire through interviewer-administered data collection, where data collectors asked the 26 drug–drug interaction knowledge questions, followed by the remaining survey sections, and recorded responses electronically. Alternatively, pharmacists who preferred self-administration completed the same questionnaire independently via an electronic link hosted on Google Forms. The questionnaire content and order were identical across both approaches, and participants were asked not to consult external references to minimize information bias.

The final questionnaire consisted of four main sections:

(1) Demographics: Included age, gender, country of graduation, academic qualification, and years of professional experience.
(2) Knowledge of DDIs (26 items): Assessed participants’ ability to identify and classify clinically relevant DDI pairs. Pharmacists were asked to classify each pair as “no interaction,” “contraindicated,” “may be used with monitoring,” or “not sure.”
(3) Awareness and Practice Behaviors: Covered awareness of DDIs, frequency of encountering DDIs in practice, approaches to identifying and managing them, communication with patients and prescribers, and resources/databases (e.g., Lexicomp, Micromedex, Medscape) used for DDI checking.
(4) Training Background: Explored prior formal or continuing education in DDIs, participation in workshops or seminars, preferred educational formats for future training, and areas in which pharmacists felt additional training was needed.

### 2.5. Ethical Approval

This study was reviewed and approved by the Standing Committee for Scientific Research Ethics at Jazan University (Approval No.: REC-47/01/1567). All participants were informed about the purpose of the study, and electronic informed consent was obtained prior to participation. Participation was voluntary, and no personally identifiable information was collected.

### 2.6. Data Analysis

Data were coded, entered, and analyzed using the Statistical Package for the Social Sciences (SPSS), version 25.0 (IBM Corp., Armonk, NY, USA). Descriptive statistics, including means, standard deviations, frequencies, and percentages, were used to summarize demographic characteristics and response distributions.

Inferential analyses were conducted to examine the relationships between pharmacists’ demographic characteristics and their knowledge of drug–drug interactions (DDIs). Independent *t*-tests were used to compare mean knowledge scores between two-group variables (e.g., gender, country of graduation), while one-way analysis of variance (ANOVA) was applied to compare mean scores across variables with more than two categories (e.g., age group, qualification, years of practice). A *p*-value of <0.05 was considered statistically significant.

## 3. Results

### 3.1. Demographics

A total of 219 community pharmacists from the Jazan Region participated in the study. The majority were male (71.7%) and aged between 20–30 years (63.9%), with 27.4% aged 31–40 years. Most held a (Doctor of Pharmacy) PharmD degree (63.9%), while 32.4% had a (Bachelor of Pharmacy) B. Pharm, and 3.7% possessed postgraduate qualifications (Table 1). Notably, 76.3% had less than 10 years of practice experience, and 62.6% were graduates of Saudi institutions (Table 1).

### 3.2. Knowledge of Drug–Drug Interactions

Pharmacists’ responses varied considerably across the 26 DDI scenarios, with each classified as either “No interaction,” “Monitoring required,” “Contraindicated,” or “Not sure.” While some combinations—such as sildenafil and isosorbide mononitrate—were correctly identified as contraindicated by 61.6% of respondents, many others were widely misclassified (Table S2). Only 43.4% correctly identified the contraindication between pimozide and ketoconazole, and 44.1% recognized the need for monitoring between phenytoin and cimetidine (Table S2).

Out of 26 interaction pairs, only sildenafil with isosorbide mononitrate was correctly identified by most (61.6%) (Table 2). The five least identified, often encountered, or risky combinations included dopamine with phenytoin (6.6%), conjugated estrogens with raloxifene (8.4%), theophylline with omeprazole (10.2%), raloxifene with alendronate (13.7%), and fexofenadine HCl, metoprolol, and diphenhydramine with warfarin (19.2%) (Table 2).

Pharmacists’ performance on the DDI knowledge assessment exhibited considerable variability. The average score was 9.63 out of 26, with a median of 9.00. Scores ranged from 0 to 22, with a standard deviation of 4.58, indicating a broad distribution of competency levels (Table S3).

Analysis of knowledge scores across demographic groups showed notable differences in DDI assessment performance. Knowledge scores generally declined with increasing age, from pharmacists aged 20–30 years (9.94 ± 4.60) and 31–40 years (9.40 ± 4.70) to those aged 41–50 years (7.06 ± 3.90), with slightly higher scores observed among pharmacists older than 50 years (8.00 ± 3.50) (Table S4). Female pharmacists demonstrated higher knowledge scores (10.74 ± 5.40) compared with male pharmacists (9.08 ± 4.20). Regarding academic qualification, pharmacists holding a PharmD degree achieved the highest mean knowledge score (10.53 ± 4.80), followed by B. Pharm graduates (8.01 ± 3.60), while those with postgraduate qualifications had the lowest scores (6.12 ± 4.20). Knowledge scores decreased with increasing years of practice, with the highest scores observed among pharmacists with less than 10 years of experience (10.14 ± 4.60) and progressively lower scores among those with 11–20 years (8.40 ± 3.80), 21–30 years (6.46 ± 4.00), and more than 30 years of practice (5.00 ± 2.20) (Table S4). Pharmacists who graduated from Saudi institutions demonstrated higher knowledge scores (10.22 ± 4.70) compared with those who graduated outside Saudi Arabia (8.44 ± 4.20), suggesting potential differences in curricular or clinical exposure (Table S4).

Knowledge scores were analyzed across key demographic groups using ANOVA and *t*-tests. Age was not significantly associated with scores (*p* = 0.106), although pharmacists aged 20–30 had the highest mean (9.94 ± 4.6), followed by those aged 31–40 years (9.40 ± 4.7) (Table 3). Those 41–50 scored lower (7.06 ± 3.9), and over 50 had a mean of 8.00 ± 3.5.

Women scored higher (10.74 ± 5.4) than men (9.08 ± 4.2, *p* = 0.016). Academic qualification was strongly linked (*p* < 0.001): PharmD holders scored the highest (10.53 ± 4.8), B. Pharm holders next (8.01 ± 3.6), and postgraduate degree holders the lowest (6.12 ± 4.2). Practice years also predicted scores (*p* = 0.002): those with less than 10 years of practice scored highest (10.14 ± 4.6), while those with 21–30 and 31–40 years of practice scored lower (6.46 ± 4.0 and 5.00 ± 2.2), indicating a possible decline in knowledge over time. Graduation location affected scores, with Saudi-educated pharmacists scoring higher (10.22 ± 4.7) than those educated abroad (8.44 ± 4.2, *p* = 0.005) (Table 3).

### 3.3. Practice Behaviors and Perceptions Related to Drug–Drug Interactions

Beyond demographic predictors and knowledge levels, the study also examined pharmacists’ perceptions, practices, and systemic influences related to the management of DDIs. These findings are organized into six thematic domains: (1) Awareness, Identification, and Detection; (2) Sources of Information and Continuing Education; (3) Communication and Counseling; (4) Systemic and Communication Barriers; (5) Guidelines and Compliance; (6) Improvements and Preferences.

#### 3.3.1. Awareness, Identification, and Detection

Pharmacists’ self-rated awareness of DDIs was generally consistent with their objective knowledge scores. Those who rated their awareness as high (7–10) had the highest average knowledge score out of 26 (10.9 ± 5.2), while those with moderate awareness (4–6) had an average score of (10.2 ± 4.3). Participants reporting low awareness (1–3) scored significantly lower (7.7 ± 3.5), indicating limited DDI recognition skills (Table S5).

Pharmacists who used patient medication history as their primary method to identify DDIs achieved the highest mean knowledge score (11.1 ± 4.9), followed by those who relied on colleagues for consultation (10.3 ± 4.1). participants who reported using drug information databases had lower average scores (9.2 ± 4.0). Respondents selecting “Other” methods had substantially lower scores (3.7 ± 2.6), potentially reflecting inconsistent or informal identification practices (Table 4).

Knowledge scores varied according to the drug interaction software tools used by pharmacists. Users of Micromedex achieved the highest average score (10.85 ± 5.39), closely followed by Lexicomp (10.47 ± 4.30) and UpToDate (10.28 ± 3.89). These three tools appear to be associated with higher DDI knowledge levels. Pharmacists relying on Medscape had lower scores (8.95 ± 4.15), and those who reported not using any software had the lowest mean score (7.33 ± 3.70) (Table 5).

#### 3.3.2. Sources of Information and Continuing Education

The choice of information sources appeared to influence the knowledge of DDI among pharmacists. Participants who use a combination of continuing education, journals, and online forums had a relatively high mean score (10.6 ± 5.35), suggesting that diversified learning may reinforce knowledge. Similarly, pharmacists who relied on continuing education or pharmaceutical journals scored consistently (10.0 ± 2.71 and 10.0 ± 3.37, respectively) (Table S6). Those who relied on online forums or communities performed lower (8.9 ± 4.28), and those selecting none of them scored markedly lower (5.6 ± 4.13), indicating a possible knowledge gap (Table S6).

Pharmacists who received training on DDIs through professional development activities achieved the highest mean knowledge score (11.34 ± 4.35), outperforming those who received training during formal education (9.30 ± 4.08) (Table 6). Participants with no prior training still performed moderately well (9.52 ± 5.43), while those uncertain about having received any DDI-related training scored the lowest (6.60 ± 3.09) (Table 6).

#### 3.3.3. Communication and Counseling

Pharmacists who employed both verbal and written communication when counseling patients on DDIs demonstrated the highest mean knowledge scores (10.9 ± 5.4), followed by those using only written materials (10.5 ± 2.8) and verbal communication alone (10.3 ± 4.0) (Table S6). Scores were lower for those using educational software or videos (8.9 ± 2.9 and 9.0 ± 5.7). Lower knowledge scores were observed among pharmacists providing additional counseling sessions (5.5 ± 3.2) or using alternative communication methods (4.0 ± 2.8), although these subgroups were small (Table S7).

#### 3.3.4. Systemic and Communication Barriers

The most commonly reported barriers to counseling patients on DDIs were limited patient understanding (31.5%) and time constraints during dispensing (29.2%) (Table S8). Other challenges included patient resistance to information (11.6%) and language or communication barriers (8.3%). A smaller proportion (6.5%) mentioned the lack of visual aids or educational materials, while 5.1% reported no significant challenges (Table S8).

The most frequently cited barriers to collaboration with healthcare providers were limited availability or responsiveness (30.6%) and a lack of time during interactions (26.9%) (Table 7). Nearly one-quarter (24.9%) of pharmacists also reported difficulty in establishing effective communication channels (Table 7).

#### 3.3.5. Guidelines and Compliance

Knowledge scores increased with greater self-reported familiarity with institutional or regulatory policies related to medication safety and drug–drug interaction management. Pharmacists who were very familiar with DDI policies had the highest mean score (10.8 ± 5.2), followed by those who were somewhat familiar (9.8 ± 4.1) (Table 8). Those who were unsure (8.8 ± 4.3) or unfamiliar (8.4 ± 3.5) scored lower (Table 8). This shows that guideline awareness correlates with DDI knowledge.

Among pharmacists following formal strategies for DDI guidelines, those conducting regular audits had the highest knowledge score (11.5 ± 5.4), followed by those using technology-based tools (11.0 ± 5.3) (Table S9). Pharmacists applying systematic processes (9.9 ± 3.8) and training (9.6 ± 4.3) also showed strong knowledge. Lower scores appeared in those citing collaboration with regulators (8.6 ± 4.4) (Table S9).

#### 3.3.6. Improvements and Preferences

Most pharmacists see increased training (39.4%) and tech tools (34.7%) as key to improving DDI awareness. Collaboration with healthcare providers (16.0%) and clearer guidelines (5.2%) were also noted (Table 9). These findings underscore the importance of ongoing education and technological support in community DDI management.

Most pharmacists preferred online courses (30.4%) and workshops (28.5%) as their top choices for future DDI training, both of which were linked to the highest average knowledge scores (10.6/26) (Table 10). Webinars were also popular (24.8%) but slightly less effective based on mean scores (9.8). Very few favored printed materials (7.5%) or showed no interest (5.1%) (Table 10).

Respondents who were not interested in future training or preferred unspecified methods had the lowest performance, indicating a strong preference for interactive and technology-supported learning methods.

## 4. Discussion

The aim of this study was to assess community pharmacists’ knowledge, attitudes, and practices regarding drug–drug interactions (DDIs) in the Jazan Region of Saudi Arabia and to identify factors associated with knowledge gaps and perceived barriers to effective DDI management. Overall, pharmacists demonstrated limited recognition of clinically significant DDIs, with knowledge scores varying according to demographic and professional characteristics, training exposure, and use of clinical decision-support tools. Commonly reported barriers included time constraints, limited prescriber accessibility, and patient-related challenges, while structured training and technology-based solutions.

The findings of this study emphasize a deficit in DDI knowledge among community pharmacists in Jazan Region. With a mean accuracy of 37%, our results are nearly identical to those from Jeddah (38.2%) [14], and the central region of Saudi Arabia, where only 5 of 26 pairs were correctly identified by most participants [15]. Such findings also align with Lebanon (mean 5.62/15) [24] and Ethiopia, where major contraindications were frequently misclassified [25]. In contrast, higher overall knowledge was reported in Egypt (58.3%) [22], possibly reflecting structured continuing education and institutional emphasis on clinical safety. Collectively, these data indicate that while knowledge remains inconsistent across contexts, the Saudi performance in both Jeddah and Jazan mirrors wider regional challenges.

Specific interactions, such as sildenafil–isosorbide mononitrate, was the most reliably recognized interaction (61.6%) among participants, in line with regional studies where it consistently scored highest (74.6% in central region of Saudi Arabia; 78.8% in Egypt) [15,22]. However, other high-risk interactions mediated by CYP3A4 (e.g., simvastatin–itraconazole, pimozide–ketoconazole) were not consistently recognized. For example, Egyptian pharmacists reported an accuracy rate of 66.6% for simvastatin–itraconazole [22], whereas our cohort achieved substantially lower rates. Furthermore, our very low recognition of dopamine–phenytoin (6.6%) and theophylline–omeprazole (10.2%) parallels Lebanon [24] and Indonesia [23], where less “classic” interactions were often missed. This pattern suggests selective familiarity: pharmacists recognize widely emphasized, life-threatening interactions but often overlook more mechanistically complex or monitoring-dependent combinations, which may still contribute substantially to preventable adverse drug events.

There were significant differences across demographic and educational factors. Female pharmacists, PharmD holders, those who graduated from Saudi Arabia, and those with fewer than 10 years of experience scored higher. Similar trends were reported in the study that was conducted in Jeddah, where both gender and years of practice predicted higher knowledge [14] and in Indonesia, where female pharmacists showed greater awareness than males [23]. Egypt also highlighted proactive DDI-checking behaviors as a positive predictor [22], while Lebanon reported a U-shaped curve where very recent and very senior pharmacists outperformed mid-career peers, possibly reflecting knowledge decay without reinforcement [24]. However, similar studies conducted in the central region of Saudi Arabia and Sudan reported no demographic differences [15,26]. Furthermore, pharmacists who received DDIs training through professional development activities outperformed those who received training only during formal undergraduate education. This is consistent with Egypt, where structured continuing education was a key predictor of DDI competence [22]. On the other hand, reliance on ad hoc resources such as informal mobile apps, as reported in Lebanon, was associated with poorer outcomes [24]. These comparisons highlight the need to embed DDIs into both undergraduate curricula and post-licensure professional development to ensure sustained competence.

Self-rated awareness could be correlated with performance, and that pharmacists who routinely reviewed medication histories, consulted colleagues, or encountered DDI cases performed better. This suggests that reflective practice and active engagement reinforce competence. In comparison, Sudanese pharmacists reported high vigilance, with 87.2% routinely screening prescriptions [26], whereas Ethiopian pharmacists displayed favorable attitudes but limited implementation [25]. Therefore, our results highlight the importance of transforming awareness into structured, repeatable practice, supported by both personal initiative and institutional mechanisms.

Pharmacists who relied on robust clinical decision-support software, such as Micromedex, Lexicomp, and UpToDate, achieved significantly higher DDI knowledge scores than those using Medscape or no software at all. This finding is supported by several comparative studies demonstrating substantial variability in the accuracy, sensitivity, and comprehensiveness of commonly used DDI databases and screening tools. For example, evaluations comparing multiple DDI software programs have shown that Lexi-Interact and Micromedex consistently outperform other platforms in detecting clinically important interactions, while lower agreement and reduced detection rates have been reported for less comprehensive tools [27]. Similar variability has been observed across different therapeutic areas, reinforcing that reliance on a single or non-curated database may lead to missed or misclassified interactions. These findings parallel data from Lebanon, where reliance on non-curated tools such as Drugs.com was associated with weaker DDI knowledge [24], and contrast with evidence from Indonesia, where high adherence to DDI screening (≈90%) was achieved largely through institutional mandates requiring accredited decision-support systems [23]. Together, these data support the notion that pharmacists’ use of different DDI resources can lead to variation in interaction recognition and underscore the value of integrated, high-performance decision support.

Barriers were raised by pharmacists that could negatively impact effective DDI communication, including time constraints, limited prescriber accessibility, and patient comprehension challenges. Similar obstacles were identified in Lebanon [24] and Ethiopia [25] while Sudanese pharmacists displayed stronger assertiveness, with over 60% refusing to dispense unsafe prescriptions [26]. Institutionalized hospital protocols, such as those in Indonesia, have mitigated such challenges by standardizing pharmacist–physician communication pathways [23]. These comparisons suggest that local pharmacists’ willingness to act is insufficient without systemic reinforcement through policy and workflow redesign. In addition, workflow interruptions, prescriber inaccessibility, and limited patient understanding remain recurrent systemic barriers. Similar findings were reported in Lebanon, where workload and time constraints hindered practice; Ethiopia, where lack of standardized protocols was a limiting factor; and Sudan, where prescriber resistance was common [24,25,26]. Additionally, a qualitative study from Canada reported that community pharmacists face substantial barriers to effective DDI assessment, including limited access to patient clinical information, high workload pressures, and alert fatigue associated with computerized decision-support systems. These challenges contributed to missed or deprioritized alerts, despite pharmacists’ awareness of their professional responsibility in preventing DDIs [11]. These barriers highlight the importance of institutional governance—through audits, protocols, and compliance monitoring—in enabling pharmacists to effectively operationalize their knowledge.

Facilitators that were thought to enhance pharmacist awareness and knowledge, such as familiarity with guidelines and participation in institutional compliance strategies, such as audits and electronic alerts, were strongly associated with higher DDI knowledge. This aligns with Indonesian hospitals, where strong governance frameworks translated into high adherence to screening protocols [23]. Not only that, but also emphasizing the pharmacists’ own calls for structured training (38%) and advanced technology (34%) as top priorities for improvement. These align with regional calls: Lebanon for structured Continuing Professional Development (CPD), Egypt for integration of point-of-care tools, and Indonesia for enhanced interprofessional collaboration [22,23,24]. Collectively, this indicates that beyond individual competence, systemic enablers are central to consistent and safe DDI management.

Although the use of clinical decision-support software was associated with higher DDI knowledge in our study, reliance on software alone may be insufficient to ensure consistent detection of clinically significant drug–drug interactions. Evidence from a U.S. performance evaluation of community pharmacy DDI software demonstrated that commonly used systems failed to detect approximately one-third of clinically important interactions, with substantial variability in sensitivity across platforms and installations [28]. These findings suggest that while technology can support pharmacists’ decision-making, it cannot fully compensate for gaps in clinical judgment or pharmacological knowledge. Consequently, CPD and structured training remain essential to reinforce pharmacists’ ability to critically interpret alerts, recognize high-risk interactions, and apply appropriate clinical management beyond automated recommendations.

These findings emphasize the importance of direct clinical interaction and interdisciplinary communication in fostering a deeper understanding of DDI awareness. They also indicate a positive link between the use of advanced tools and increased DDI awareness. The results support encouraging consistent use of specialized clinical software in pharmacy practice to enhance decision-making accuracy. Additionally, these findings suggest that ongoing professional education may be more effective in improving practical DDI knowledge than initial academic exposure alone. They highlight that most barriers to patient counseling stem from communication dynamics and workflow constraints rather than knowledge gaps, emphasizing the need for patient education resources and sufficient time to improve pharmacist–patient interactions in community pharmacy settings. Furthermore, these findings point to persistent communication challenges that could hinder interprofessional collaboration in managing drug–drug interactions. Overall, they underscore the importance of continuous education and technological support in community DDI management.

These findings are particularly important in the evolving healthcare landscape of Saudi Arabia. The government’s increasing reliance on the *Wasfaty* e-prescription program has expanded the clinical role of community pharmacists, enabling them to dispense a wider range of prescription medications that were previously limited to hospital pharmacies. This transformation aligns with the national Vision 2030 and Health Sector Transformation Program, which emphasize accessibility, digitalization, and continuity of care. As *Wasfaty* bridges governmental healthcare services with community-based dispensing, community pharmacists are becoming integral to ensuring medication safety, particularly through the identification and management of drug–drug interactions. Therefore, assessing and strengthening their knowledge and practices is timely and essential to optimize patient outcomes and support the ongoing advancement of pharmaceutical care in Saudi Arabia.

## 5. Limitations

The knowledge assessment was limited to a select number of drug pairs, which may not fully capture pharmacists’ overall competence in identifying DDIs across therapeutic classes.

Additionally, the study was conducted exclusively in the Jazan Region, which may limit generalizability to other parts of Saudi Arabia with different practice environments or healthcare infrastructures. Nevertheless, comparisons with similar national and international studies comprehensively addressed the discussion.

## 6. Conclusions

This study shows gaps in community pharmacists’ knowledge and practices on DDIs in Jazan. Globally, pharmacists recognize DDIs but do not always act. Urgent actions include adding DDI training to education, continuing education, and electronic DDI-check systems. Closing these gaps can improve pharmacists’ role in preventing adverse drug events and ensure safer medication use. The research may prompt stakeholders to develop tools, including AI, to improve pharmacists’ knowledge and patient safety.

## Figures and Tables

**Table 1:** Demographic and Professional Characteristics of Community Pharmacists (n = 219).

	Category	n	%
**Age**	20–30	140	63.9
	31–40	60	27.4
	41–50	16	7.4
	>50	3	1.4
**Gender**	Male	157	71.7
	Female	62	28.3
**Academic Qualification**	B. Pharm	71	32.4
	PharmD	140	63.9
	Postgraduate	8	3.7
**Years of Practice**	<10 years	167	76.3
	10–20 years	35	16.0
	21–30 years	13	5.9
	31–40 years	4	1.8
**Country of Graduation**	Saudi Arabia	137	62.6
	Outside Saudi Arabia	82	37.4

**Table 2:** Correct Response Rates for Each Drug Pair Evaluated in the Knowledge Assessment.

Drug–Drug Interaction Pair	Correct n (%)	Incorrect n (%)
Warfarin and cimetidine	69 (32.1)	146 (67.9)
Sildenafil and isosorbide mononitrate	133 (61.6)	83 (38.4)
Conjugated estrogens and raloxifene	18 (8.4)	197 (91.6)
Fexofenadine HCL and metoprolol	41 (19.2)	173 (80.8)
Theophylline and ciprofloxacin	98 (46.0)	115 (54.0)
Pimozide and ketoconazole	92 (43.4)	120 (56.6)
Methyldopa and phenobarbital	100 (46.7)	114 (53.3)
Phenytoin and cimetidine	94 (44.1)	119 (55.9)
Itraconazole and quinidine	124 (57.9)	90 (42.1)
Amiodarone and simvastatin	109 (50.9)	105 (49.1)
Methotrexate and probenecid	95 (44.8)	117 (55.2)
Diphenhydramine and warfarin	41 (19.3)	171 (80.7)
Raloxifene and alendronate	29 (13.7)	183 (86.3)
Warfarin and diflunisal	81 (38.0)	132 (62.0)
Amiodarone and fluconazole	91 (44.0)	116 (56.0)
Theophylline and omeprazole	22 (10.2)	193 (89.8)
Sulfinpyrazone and warfarin	73 (34.4)	139 (65.6)
Meperidine and phenelzine	91 (43.3)	119 (56.7)
Fluconazole and phenytoin	110 (51.6)	103 (48.4)
Warfarin and nortriptyline	110 (51.9)	102 (48.1)
Amoxicillin and acetaminophen with codeine	97 (45.8)	115 (54.2)
Digoxin and clarithromycin	92 (43.0)	122 (57.0)
Cyclosporine and rifampicin	87 (40.7)	127 (59.3)
Alprazolam and itraconazole	82 (38.3)	132 (61.7)
Dopamine and phenytoin	14 (6.6)	199 (93.4)
Ciprofloxacin and tizanidine	116 (54.2)	98 (45.8)

**Table 3:** Association Between Demographic Variables and Knowledge Scores.

Variable	Demographic Group	n	Knowledge Score (Mean (/26) ± SD)	*p*-Value
**Age**	**20–30**	140	9.94 ± 4.6	0.106
	**31–40**	60	9.40 ± 4.7	
	**41–50**	16	7.06 ± 3.9	
	**More than 50 years**	3	8.00 ± 3.5	
**Gender**	**Female**	62	10.74 ± 5.4	0.016 *
	**Male**	157	9.08 ± 4.2	
**Pharmacy Academic Qualification**	**B. Pharm**	71	8.01 ± 3.6	<0.001 *
	**PharmD**	140	10.53 ± 4.8	
	**Postgraduate studies**	8	6.12 ± 4.2	
**Years of Practice**	**less than 10 years**	167	10.14 ± 4.6	0.002 *
	**10–20**	35	8.40 ± 3.8	
	**30–21**	13	6.46 ± 4.0	
	**40–31**	4	5.00 ± 2.2	
**Country of Graduation**	**Saudi Arabia**	137	10.22 ± 4.7	0.005 *
	**Outside Saudi Arabia**	82	8.44 ± 4.2	

Values are mean ± SD. *t*-test and one-way ANOVA were used; *p* < 0.05 was considered significant (*).

**Table 4:** Knowledge Scores by Method Used to Identify Drug–Drug Interactions.

Identification Method	Respondents	Mean Score (Mean (/26) ± SD)
Patient’s medication history	66	11.1 ± 4.9
Consultation with colleagues	56	10.3 ± 4.1
Drug information databases	87	9.2 ± 4.0
Other	7	3.7 ± 2.6

**Table 5:** Knowledge Scores by Type of Drug Interaction Software Tool Used.

Software Tool	Respondents (n)	Mean Score (Mean (/26) ± SD)
Micromedex	54	10.85 ± 5.39
Lexicomp	38	10.47 ± 4.30
UpToDate	50	10.28 ± 3.89
Other	3	9.33 ± 4.16
Medscape	58	8.95 ± 4.15
I don’t use any	12	7.33 ± 3.70

**Table 6:** Knowledge Scores by Source of Training on Drug–Drug Interactions.

Training Received	Respondents (n)	Mean Score (Mean (/26) ± SD)
Yes, through professional development	77	11.34 ± 4.35
No	25	9.52 ± 5.43
Yes, during education	98	9.30 ± 4.08
Not Sure	15	6.60 ± 3.09

**Table 7:** Reported Challenges in Collaborating and Communication with Healthcare Providers on Drug–Drug Interactions.

Reported Challenge	Respondents (n)	Percentage (%)
Limited availability or responsiveness of healthcare providers	59	30.6
Lack of time during healthcare provider interactions	52	26.9
Difficulty in establishing effective communication channels	48	24.9
Differences in interpretation or prioritization of drug interactions	17	8.8
Limited awareness or interest from healthcare providers	17	8.8

**Table 8:** Knowledge Scores by Level of Familiarity with Drug–Drug Interaction Guidelines.

Familiarity Level	Respondent (n)	Mean Score (Mean (/26) ± SD)
Very familiar	71	10.8 ± 5.2
Somewhat familiar	98	9.8 ± 4.1
Not sure	18	8.8 ± 4.3
Not familiar	27	8.4 ± 3.5

**Table 9:** Suggested Improvements to Enhance Pharmacist Awareness of Drug–Drug Interactions.

Suggested Improvement	Respondents (n)	Percentage (%)
More training opportunities	84	39.4
Improved technology tools	74	34.7
Better collaboration with healthcare providers	34	16
Clearer guidelines and policies	11	5.2
Other	10	4.7

**Table 10:** Knowledge Scores by Preferred Future Training Method.

Training Preference	Respondents (n)	Percentage of Respondents (%)	Mean Score (Mean (/26) ± SD)
Online courses	65	30.4	10.6 ± 5.0
Workshops	61	28.5	10.6 ± 4.1
Webinars	53	24.8	9.8 ± 4.1
Printed materials	16	7.5	9.1 ± 3.5
Not interested in future training	11	5.1	6.7 ± 4.5
Other	8	3.7	5.4 ± 3.0

## Data Availability

The data presented in this study are available on request from the corresponding author due to privacy and ethical restrictions.

## Supplementary Materials

The following supporting information can be downloaded at: https://www.mdpi.com/article/10.3390/pharmacy14010012/s1, File S1: Questionnaire; Table S1: STROBE Statement—Checklist of items that should be included in reports of cross-sectional studies; Table S2: Overall Knowledge Scores on Drug-Drug Interactions Among Participants; Table S3: Descriptive Statistics for Drug-Drug Interaction Knowledge Scores Among Community Pharmacists; Table S4: Knowledge Scores by Demographic Characteristics; Table S5: Knowledge Scores by Self-Rated Awareness of Drug–Drug Interactions; Table S6: Knowledge Scores by Primary Source of Drug–Drug Interaction Information; Table S7: Knowledge Scores by Communication Method Used When Counseling Patients About Drug–Drug Interactions; Table S8: Reported Barriers to Patient Counseling on Drug–Drug Interactions; Table S9: Knowledge Scores by Strategy Used to Ensure Compliance with DDI Guidelines.
