# Supplementary material for: Drug-Drug Interaction Knowledge, Practices, and Barriers in Community Pharmacies: A Cross-Sectional Study from Jazan Region, Saudi Arabia
Source: Pharmacy (Basel). 2026 Jan 23;14(1):12. doi: 10.3390/pharmacy14010012 (PMC12921910; doi:10.3390/pharmacy14010012)
Supplement: Supplementary file 1 [file pharmacy-14-00012-s001.zip › pharmacy-4031606-supplementary1-Questionnaire.pdf]

Supplementary Materials File S1.

Questionnaire

Demographic Characteristics

1. What is your age group?

- 20–30 years
- 31–40 years
- 41–50 years
- >50 years

2. What is your gender?

- Male
- Female

3. What is your highest academic qualification in pharmacy?

- B.Pharm
- PharmD
- Postgraduate degree

4. How many years of professional practice do you have?

- <10 years
- 10–20 years
- 21–30 years
- 31–40 years

5. Where did you obtain your pharmacy degree?

- Saudi Arabia
- Outside Saudi Arabia

Drug–Drug Interaction Knowledge Assessment

Instruction: For each drug pair, select the most appropriate classification.

Drug–Drug Interaction Pair	No interaction	Requires monitoring	Contraindicated	Not sure
Warfarin + Cimetidine				
Sildenafil + Isosorbide mononitrate				
Conjugated estrogens + Raloxifene				
Fexofenadine HCl + Metoprolol				
Theophylline + Ciprofloxacin				
Pimozide + Ketoconazole				

Methyldopa + Phenobarbital				
Phenytoin + Cimetidine				
Itraconazole + Quinidine				
Amiodarone + Simvastatin				
Methotrexate + Probenecid				
Diphenhydramine + Warfarin				
Raloxifene + Alendronate				
Warfarin + Diflunisal				
Amiodarone + Fluconazole				
Theophylline + Omeprazole				
Sulfinpyrazone + Warfarin				
Meperidine + Phenelzine				
Fluconazole + Phenytoin				
Warfarin + Nortriptyline				
Amoxicillin + Acetaminophen/Codeine				
Digoxin + Clarithromycin				
Cyclosporine + Rifampicin				
Alprazolam + Itraconazole				
Dopamine + Phenytoin				
Ciprofloxacin + Tizanidine				

Awareness, Information Sources, and Communication Practices

What is your primary source of information for drug–drug interactions? *(Select one or more options)*

☐ Continuing education

☐ Pharmaceutical journals

☐ Online forums or professional communities

☐ All of the above

☐ None of the above

☐ Other (please specify): ___________

Which drug–drug interaction software tool do you primarily use in your practice?
(Select one option)

- Micromedex
- Lexicomp
- UpToDate
- Medscape
- Other (please specify): ___________
- I do not use any drug–drug interaction software

What is your primary source of information for drug–drug interactions?
(Select one option)

- Continuing education
- Pharmaceutical journals
- Online forums or professional communities
- All of the above
- None of the above
- Other (please specify): ___________

Have you received any formal training on drug–drug interactions?
(Select one option)

- Yes, through professional development activities
- Yes, during formal education
- No
- Not sure

What method do you primarily use to communicate potential drug–drug interactions to patients?
(Select one option)

- Both verbal and written communication
- Written materials (e.g., leaflets, brochures)
- Verbal communication
- Educational software or videos
- Rely on healthcare providers to communicate this information
- Provide additional counseling sessions
- Other (please specify): ___________

Reported Barriers to Patient Counseling on Drug–Drug Interactions

What barriers do you most commonly face when counseling patients about drug–drug interactions?
(Select one option)

- Limited patient understanding
- Time constraints during dispensing
- Patient resistance to information
- Language or communication barriers
- Lack of visual aids or educational materials
- No significant challenges
- Not sure

- Other (patient-related challenges; please specify): ___________

Guidelines and Compliance

How familiar are you with drug–drug interaction–related guidelines or policies in your practice?
(Select one option)

- Very familiar
- Somewhat familiar
- Not sure
- Not familiar

Which strategy do you primarily use to ensure compliance with drug–drug interaction (DDI) guidelines or policies in your practice?
(Select one option)

- Regular audits
- Technology tools with built-in compliance features
- Systematic processes to identify and manage DDIs
- Training sessions for the pharmacy team
- Collaboration with regulatory affairs specialists
- Not sure
- Other compliance measures (please specify): ___________

Improvements and Preferences

What improvements do you believe would most enhance pharmacists' awareness of drug–drug interactions?
(Select one option)

- More training opportunities
- Improved technology tools
- Better collaboration with healthcare providers
- Clearer guidelines and policies
- Other (please specify): ___________
