# Supplementary material for: Drug-Drug Interaction Knowledge, Practices, and Barriers in Community Pharmacies: A Cross-Sectional Study from Jazan Region, Saudi Arabia
Source: Pharmacy (Basel). 2026 Jan 23;14(1):12. doi: 10.3390/pharmacy14010012 (PMC12921910; doi:10.3390/pharmacy14010012)
Supplement: Supplementary file 1 [file pharmacy-14-00012-s001.zip › pharmacy-4031606-supplementary3-Tables S2-S9.pdf]

Supplementary Materials

- **Descriptive Statistics for Drug-Drug Interaction Knowledge Scores Among Community Pharmacists**

Table S2. Overall Knowledge Scores on Drug-Drug Interactions Among Participants.

Drug–drug interaction pair	No interaction n (%)	Monitoring n (%)	Contraindication n (%)	Not sure n (%)	Total Responses
Warfarin and cimetidine	6 (2.8%)	69 (32.1%)	125 (58.1%)	15 (7.0%)	215
Sildenafil and isosorbide mononitrate	3 (1.4%)	66 (30.6%)	133 (61.6%)	14 (6.5%)	216
Conjugated estrogens and raloxifene	18 (8.4%)	77 (35.8%)	101 (47.0%)	19 (8.8%)	215
Fexofenadine HCL and metoprolol	41 (19.2%)	88 (41.1%)	62 (29.0%)	23 (10.7%)	214
Theophylline and ciprofloxacin	9 (4.2%)	98 (46.0%)	87 (40.8%)	19 (8.9%)	213
Pimozide and ketoconazole	12 (5.7%)	89 (42.0%)	92 (43.4%)	19 (9.0%)	212
Methyldopa and phenobarbital	14 (6.5%)	100 (46.7%)	77 (36.0%)	23 (10.7%)	214
Phenytoin and cimetidine	6 (2.8%)	94 (44.1%)	93 (43.7%)	20 (9.4%)	213
Itraconazole and quinidine	10 (4.7%)	65 (30.4%)	124 (57.9%)	15 (7.0%)	214
Amiodarone and simvastatin	18 (8.4%)	109 (50.9%)	71 (33.2%)	16 (7.5%)	214
Methotrexate and probenecid	5 (2.4%)	95 (44.8%)	87 (41.0%)	25 (11.8%)	212
Diphenhydramine and warfarin	41 (19.3%)	83 (39.2%)	62 (29.2%)	26 (12.3%)	212
Raloxifene and alendronate	29 (13.7%)	97 (45.8%)	64 (30.2%)	22 (10.4%)	212
Warfarin and diflunisal	13 (6.1%)	81 (38.0%)	91 (42.7%)	28 (13.1%)	213
Amiodarone and fluconazole	16 (7.7%)	91 (44.0%)	77 (37.2%)	23 (11.1%)	207
Theophylline and omeprazole	22 (10.2%)	95 (44.2%)	75 (34.9%)	23 (10.7%)	215

Sulfinpyrazone and warfarin	14 (6.6%)	73 (34.4%)	98 (46.2%)	27 (12.7%)	212
Meperidine and phenelzine	11 (5.2%)	84 (40.0%)	91 (43.3%)	24 (11.4%)	210
Fluconazole and phenytoin	11 (5.2%)	110 (51.6%)	73 (34.3%)	19 (8.9%)	213
Warfarin and nortriptyline	13 (6.1%)	110 (51.9%)	73 (34.4%)	16 (7.5%)	212
Amoxicillin and acetaminophen with codeine	97 (45.8%)	52 (24.5%)	44 (20.8%)	19 (9.0%)	212
Digoxin and clarithromycin	11 (5.1%)	92 (43.0%)	89 (41.6%)	22 (10.3%)	214
Cyclosporine and rifampicin	21 (9.8%)	87 (40.7%)	86 (40.2%)	20 (9.3%)	214
Alprazolam and itraconazole	21 (9.8%)	82 (38.3%)	95 (44.4%)	16 (7.5%)	214
Dopamine and phenytoin	14 (6.6%)	89 (41.8%)	96 (45.1%)	14 (6.6%)	213
Ciprofloxacin and tizanidine	14 (6.5%)	70 (32.7%)	116 (54.2%)	14 (6.5%)	214

Table S3. Descriptive Statistics for Drug-Drug Interaction Knowledge Scores Among Community Pharmacists

Statistic	Knowledge Score
**Minimum**	0
**Maximum**	22
**Mean**	9.63
**Median**	9.00
**Standard Deviation**	4.58

Table S4. Knowledge Scores by Demographic Characteristics

Demographic Group	Category	count	mean	std	min	median	max
**Age**	20-30	140	9.94	4.6	0	10	22
	31–40	60	9.40	4.7	0	9	20
	41–50	16	7.06	3.9	2	6.5	15
	More than 50 years	3	8.00	3.5	6	6	12
**Gender**	Female	62	10.74	5.4	0	10	22
	Male	157	9.08	4.2	0	9	20

**Pharmacy academic qualification**	B.Pharm	71	8.01	3.6	0	8	16
	PharmD	140	10.53	4.8	0	10	22
	Postgraduate studies	8	6.12	4.2	0	5	14
**Years of practice**	less than 10 years	167	10.14	4.6	0	10	22
	20–11	35	8.40	3.8	0	8	16
	30–21	13	6.46	4.0	0	6	14
	40–31	4	5.00	2.2	2	5.5	7
**Country of Graduation**	Out of Saudi Arabia	82	8.44	4.2	0	8	19
	Saudi Arabia	137	10.22	4.7	0	10	22

- **Knowledge Scores by Self-Rated Awareness of Drug–Drug Interactions**

Table S5. Knowledge Scores by Self-Rated Awareness of Drug–Drug Interactions

Awareness Rating	Respondents	Mean Score (/26)	Std. Dev.
**Low awareness (1–3)**	36	7.7	3.5
**Moderate (4–6)**	88	10.2	4.3
**High awareness (7–10)**	61	10.9	5.2
**Not sure**	25	10.4	3.9
**Prefer not to answer**	5	7.0	2.3

Table S6. Knowledge Scores by Primary Source of Drug–Drug Interaction Information

Information Source	Respondents	Average Score (/26)	Std. Dev.
**Continuing education**	39	10.0	2.71
**Pharmaceutical journals**	42	10.0	3.37
**Online forums/communities**	35	8.9	4.28
**All of the above**	85	10.6	5.35
**None of the above**	9	5.6	4.13
**Other**	6	11.0	6.07

Table S7. Knowledge Scores by Communication Method Used When Counseling Patients About Drug–Drug Interactions

Communication Method	Respondents	Average Score (/26)	Std. Dev.
**Both verbal and written**	62	10.9	5.4
**Written materials (e.g., leaflets, brochures)**	27	10.5	2.8

**Verbal communication**	80	10.3	4.0
**Utilize educational software or videos**	17	8.9	2.9
**Rely on healthcare providers**	12	9.0	5.7
**Provide additional counseling sessions**	15	5.5	3.2
**Other**	2	4.0	2.8

- **Reported Barriers to Patient Counseling on Drug–Drug Interactions**

Table S8. Reported Barriers to Patient Counseling on Drug–Drug Interactions

**Challenge**	**Respondents (n)**	**Percentage (%)**
**Limited patient understanding**	68	31.5
**Time constraints during dispensing**	63	29.2
**Patient resistance to information**	25	11.6
**Language or communication barriers**	18	8.3
**Lack of visual aids or educational materials**	14	6.5
**Not sure**	13	6.0
**No significant challenges**	11	5.1
**Other (patient-related challenges)**	4	1.9

Table S9. Knowledge Scores by Strategy Used to Ensure Compliance with DDI Guidelines

**Compliance Strategy**	**Respondents**	**Average Score (/26)**	**Std. Dev.**
**Regular audits**	11	11.5	5.4
**Technology tools with built-in compliance**	40	11.0	5.3
**Systematic processes to identify/manage DDIs**	57	9.9	3.8
**Training sessions for the pharmacy team**	69	9.6	4.3
**Not sure**	23	9.3	4.8
**Collaboration with regulatory affairs specialists**	13	8.6	4.4
**Other compliance measures**	3	6.3	5.7
